# Dunham Medical College

**Published:** 1897-09

**Authors:** 


### DUNHAM MEDICAL COLLEGE.

The Editor has been requested to publish the following letter to correct a prevalent misapprehension due to irresponsible statements:

(Copy.)

### STATE BOARD OF MEDICAL REGISTRATION AND EXAMINATION.

Indianapolis, Ind., August 20th, 1897.

C. S. Fahnestock, M. D., Dean of Dunham Medical College, Chicago.

Dear Doctor:—Replying to your favor of the 19th, will say the Dunham Medical College, of Chicago, Ill., is in good standing with and fully recognized by this Board, all reports to the contrary notwithstanding.

Very truly,

(Signed) Wm. F. Curryer, M. D.,

*Secretary.*

